# The need for a One Health approach for influenza surveillance

**DOI:** 10.1016/S2214-109X(22)00240-6

**Published:** 2022-06-13

**Authors:** Siddhartha Saha, William W Davis

**Affiliations:** Influenza Division, CDC India Office, US Embassy, New Delhi-110021, India

Humidity is a major driver of influenza circulation, and this bimodal relationship is mediated by temperature.^1^ In temperate regions, influenza circulates predominantly in well-defined annual epidemics during cold months with low humidity, with very low circulation during the rest of the year.^2^ Conversely, in warmer tropical regions, there is wider variation in influenza seasonality, with year-round circulation and one or more peaks per year that also can occur in times of higher humidity.^2,3^ In south Asia, for example, influenza is known to have distinct seasonality in tropical and subtropical regions, with year-round circulation and peaks during rainy seasons.^4^ Even within tropical countries, influenza seasonality might vary, as seen in studies in China, India, and Brazil.^5–7^ WHO makes biannual influenza vaccine recommendations, timed so that vaccines can be introduced before the influenza seasons in the northern and southern hemispheres. However, variable circulation in tropical climates poses challenges for influenza vaccination timing;^3^ more granular data are needed to inform these decisions.

Therefore, the subregional analysis of influenza seasonality in Bangladesh, reported in *The Lancet Global Health* by Isha Berry and colleagues,^8^ is important to understand influenza circulation more clearly, which could inform vaccination timing and other influenza control measures in Bangladesh. The authors found that seasonal influenza activity peaks in June and July, and this peak occurs earlier in the two most densely populated metropolitan areas, followed by spatial diffusion throughout the rest of the country. The data used were from a robust sentinel surveillance system, which included over 8700 human influenza cases from 32 sentinel surveillance sites over 10 years. The findings could inform seasonal influenza vaccine timing and perhaps serve as an early warning system for rural areas for seasonal influenza epidemics. The Article also underscores the importance of systematic sentinel surveillance data with geographical representation collected for years to enable robust time-series analysis, as influenza activity is also known to vary from year to year.

The authors repeated the seasonality analysis with environmental surveillance data from live bird markets in Dhaka to examine the seasonality of avian influenza. They found that avian influenza circulated year-round, with a slight peak in April, which was different timing from the human influenza peak. These results suggest that drivers of avian influenza circulation in poultry might be different from those that drive circulation in humans. This difference was not explored in the paper, although further exploration might inform better control measures. Although the seasonality of human and avian influenza outbreaks did not coincide, the study provides important insights into understanding the year-round circulation of both seasonal and avian influenza viruses in this region.

The Article also raises an important question of variable seasonality of avian influenza in Bangladesh. Global data since 2005 on avian influenza outbreaks in poultry show the peak activity in February.^9^ Data from 2007–12 that included sampling from live poultry in Bangladesh showed peak activity from October to March, with decreased activity from April to September.^10^ Berry and colleagues identified a peak in April, which is not completely aligned with previous reports. They also reported a decline in the proportion of environmental specimens testing positive for avian influenza viruses over the 3 years of the study period. It would be interesting to evaluate the reasons for this decline; this might require data from other avian influenza surveillance that covers more years and includes samples from live poultry; such data are available for Bangladesh.^11^

Influenza type A viruses continue to pose a substantial threat to global health because of their pandemic potential through genetic reassortment with zoonotic influenza viruses. In the last three decades, human infections of avian influenza A(H5N1) and A(H7N9) viruses have caused over 1500 cases and 600 deaths.^12^ In many south Asian countries, the risk of such zoonotic infection is increasing, with increasing human and poultry population numbers and movements, and poor biosafety and biosecurity practices in backyard poultry farms and in markets.^13^ Tackling this issue requires increased collaboration and regular coordination between human and veterinary health sectors, with exchange of surveillance data and joint outbreak investigations, drawing upon the expertise of both the sectors. However, such multisectoral collaboration might need organisational changes, availability of resources, and enabling policy. Policy to address avian influenza risks in Bangladesh has advanced over the past decade, but there is more work to be done.^11^ Therefore, in Bangladesh and elsewhere, it is important to adopt and strengthen a One Health approach to influenza surveillance to continue to explore the epidemiology of human and avian influenza circulation and risks.
